# National Eye Institute Visual Function Scale in Type 2 Diabetes Patients

**DOI:** 10.1155/2016/1549318

**Published:** 2016-02-21

**Authors:** Sezen Akkaya, Sinay Düzova, Özlem Şahin, Haluk Kazokoğlu, Tayfun Bavbek

**Affiliations:** ^1^Fatih Sultan Mehmet Education and Training Hospital, Üst Bostancı, 34752 Istanbul, Turkey; ^2^Türkan Saylan Hospital, Ataşehir, 34455 Istanbul, Turkey; ^3^Marmara Üniversity, Pendik, 34978 Istanbul, Turkey; ^4^Dünya Göz Hospital, Altunizade, 34662 Istanbul, Turkey

## Abstract

*Aim*. To examine subscale and total scores of NEI-VFQ questionnaire of type 2 diabetes patients at different diabetic retinopathy (DRP) stages.* Methods*. A total number of 201 patients have been included. Prior to ophthalmological examination all patients participated in the NEI-VFQ questionnaire. The patients were divided into 5 groups according to the International Clinical Diabetic Retinopathy Disease Severity Scale (ICDRS).* Results*. The diabetes duration in general health scores (*p* = 0.029); the stage (*p* = 0.011); and clinically significant macular edema (CSME) (*p* = 0.019) in general vision were found to be the most efficient factors. In near vision activities the most efficient factors were near vision acuity (NVA) (*p* = 0.0001) and DRP stage (*p* = 0.020). EDTRS visual acuity was found to be the most efficient factor in vision specific role difficulties (*p* = 0.034) and dependency (*p* = 0.011) whereas Snellen visual acuity was found to be among the most effective factors in distance activities (DA) (*p* = 0.014) and total scores (*p* = 0.026).* Discussion*. Difference was based not on the diabetes duration, clinically significant cataract (CSCat), CSME presence, and DRP stage but on the* visual acuity* levels of the better seeing eye of the patients.

## 1. Introduction

Although visual acuity is an important criterion in order to measure a multidimensional function like vision, it still cannot enlighten us enough to understand completely how the life styles of the patients are affected from the visual function of said patients. Visual acuity will not be sufficient to define conditions such as emotionality depending on visual impairment, feeling of being incompetent, and social function loss. For this reason, several surveys have been developed in the recent years in order to investigate the effects of visual impairment to daily life [1]. American National Eye Institute Visual Function Scale (NEI-VFQ 25) is a questionnaire that has been developed in order to reach said aim [2]. The Turkish translation of NEI-VFQ 25 scale and its validity and reliability have been shown [3, 4].

Diabetic retinopathy is the primary reason for newly developed blindness in developed countries [5]. Even in cases where retinopathy does not lead to decrease in visual acuity, the function of sight is affected in different ways and the living quality of patients can decrease [6–9]. The clinical usage soundness of NEI-VFQ 25 scale in ophthalmic diseases has been confirmed and publications are present in relation to its usage on patients suffering from macular degeneration dependent on age, glaucoma, optical neuritis, dry eye, uveitis, and central vein occlusion [10–14]. However nowadays, the studies that evaluate the quality of life dependent on vision with clinical measurements of the central vision function in diabetics patients are limited [15, 16]. In our study we aimed to investigate the sub scale and total scores of NEI-VFQ questionnaire in type 2 diabetic patients in different DRP stages and the demographic and clinical characteristics that are most effective in said scores.

## 2. Subjects and Methods

201 cases that have been diagnosed with type 2 diabetes mellitus have been included in the study. This study was conducted in accordance with the tenets of the Declaration of Helsinki. The cases where the patient had Alzheimer's disease or dementia, which could prevent the patient to answer the questions, or is suffering from another disease, besides DRP and cataract, that could affect the visual functions in both eyes were not included in the study. All the cases participating in the study were informed regarding the contents of the study and letters of approval stating that they have accepted to participate in this study have been obtained from all patients.

The age, gender, duration term of diabetes (taken from the diagnosis date until the commencement date of the study), blood sugar control types (diet, oral antidiabetic, or insulin), the most recent preprandial blood glucose (values taken in the last week prior to the study), the HbA1c levels in the last month, hypertension presence, laser treatments taken previously due to DRP (panretinal, focal, and grip photocoagulation), and cataract operation histories were examined and were recorded.

Prior to ophthalmologic examinations all patients were given the NEI-VFQ. If the patients were using glasses, for example, they were advised to answer the questions of the questionnaire according to their thoughts and feelings at their time of usage of said glasses. An additional number of 13 questions were asked to all patients in addition to the 25 questions of the questionnaire and their answers were recorded into the forms.

The initial and best adjusted visual acuity for both eyes has been measured from 4 meters with Snellen and ETDRS scales. The difference between the initial visual acuity and the best adjusted visual acuity was comprised of only two sequences, the patients who did not use glasses or the patients having incompatibilities between the refraction deficiencies, and the glasses they were using were excluded from the study as it was deemed that the refraction deficiencies could affect the results of the study [17]. It has been assumed that the quality of living was in correlation with the visual acuity of the eye that had better eyesight and as a result the initial visual acuity values of eyes having better visual acuity were used [17]. During examination of near sight, the patients were asked to put their glasses on and the Jaeger scale from 35 to 40 cm was read by the patients. Those patients who did not use glasses although they needed to or those who used incompliant glasses not compliant with the refraction deficiency were excluded from the study as it was assumed that the refraction deficiencies could affect the study. The near sight acuity values that have been measured were converted to Snellen equivalent values [18].

The intraocular pressure was measured with Goldman applanation tonometry prior to biomicroscopic examination. Following this the front segment was evaluated with slit lamp biomicroscopy. The eyes of the patients were dilated with 1% tropicamide for lens examination and a drop in each eye was used. Lens examination was carried out at a 45-degree angle, with retroillumination from the pupil center with the narrowest slit lamp measure. Lens opacities classification system (LOCS II) was used separately for nuclear (NC), cortical (CC), and posterior subcapsular cataracts (PSC) in order to measure the cataract stage for both eyes [19]. The scores with stage 2 and above values were accepted to be clinically significant cataracts (CSCat) for nuclear, cortical, and posterior subcapsular cataracts [20]. The results were recorded into the forms.

The fundus examinations of the patients were carried out using a 90D Volk lens. The DRPs of the patients were scaled according to the ICDRS system [21]. According to this staging system, if there is no DRP which was accepted to be within stage 0, those within the mild NPDR (*nonproliferative diabetic retinopathy*) stages were accepted to be within stage 1, the mild NPDR stages were accepted to be included in stage 2, and the severe NPDR stage was accepted to be included in stage 3 and those within the proliferative DRP stage were included in stage 4 group (Table 1).

The patients that has received grid and/or focal laser treatment previously and the patients who did not have macula edema in their FFA and/or macula edema which was not found during their ophthalmologic examinations were included as patients with clinically significant macula edema into the study.

## 3. Results

### 3.1. Demographics Characteristics of Patients

The age and gender distribution of patients according to stages have been shown in Table 1.

There was no statistically significant difference in terms of the distribution of the age (*p* = 0.56) and gender (*p* = 0.51) groups of patients.

The number of patients suffering from hypertension was 126 (62.7%). The presence of hypertension of patients distributed according to stages has been given in Table 2.

There was no statistically significant difference between the groups in terms of hypertension distribution of patients (*p* = 0.53).

The clinical characteristics in relation to diabetes of the patients according to stages have been given in Table 3.

### 3.2. Clinical Findings of Patients

Ophthalmologic examination findings of the patients have been given in Table 4.

The near vision acuity decrease (*p* = 0.0001) results of patients according to the scales of Snellen (*p* = 0.0001) and ETDRS (*p* = 0.0001) of patients with increasing stages were statistically significant.

The difference between the average intraocular pressure values was not statistically significant (*p* = 0.77). The increase in the number of patients having had cataract surgery as DRP stage increased and the decrease in the number of patients with CSC were statistically significant (*p* = 0.01). The increase in the number of patients with CSME as DRP stages increased was significantly meaningful (*p* = 0.0001).

The NEI-VFQ-39 subscale and total scores of patients have been given in Table 5.

### 3.3. The Questionnaire Score Distribution of Patients according to Demographic Data

The questionnaire score distribution of patients according to demographic data has been shown in Table 6. As age increased between the age groups of 51–60 and 61–70, the decrease of the distance activities (*p* = 0.04), mental health (*p* = 0.04), role difficulties (*p* = 0.04), visual dependency (*p* = 0.009), and total (0.028) scores of the patients were significantly significant.

The average ocular pain scores in men were 82.47 ± 19.45 and in women were 73.30 ± 20.63; and these results were significantly higher in men in comparison to women (*p* = 0.001).

The general health scores of the group with hypertension were 44.10 ± 16.11, and the group without hypertension was 49.16 ± 16.68; the scores of the group with hypertension were significantly lower in comparison to the group without hypertension (*p* = 0.03).

### 3.4. The Questionnaire Scores Distribution of the Patients according to Clinical Characteristics

The distribution of the questionnaire scores regarding diabetes of the patients according to clinical characteristics has been shown in Table 7.

The decrease in the general vision scores as the diabetes duration increased was statistically significant (*p* = 0.007).

The decrease in general health (*p* = 0.0001), general vision (*p* = 0.0001), and near vision (*p* = 0.0001) scores as the DRP stage increased was statistically significant.

The general vision scores of the patients that used insulin to regulate blood sugar were found to be significantly lower in comparison to groups being treated with diet and OAD (oral antidiabetics) (*p* = 0.04).

### 3.5. Questionnaire Scores according to Ophthalmological Findings

The distribution of the questionnaire scores of patients according to ophthalmological findings which are shown in Table 8.

The increase in the scores of general vision (*p* = 0.0001), near activities (*p* = 0.0001), and distance activities (*p* = 0.04) as Snellen vision acuity values increased was statistically significant.

Snellen vision acuity between the groups ≤0.2 and 0.6–0.8, in terms of vision specific mental health (*p* = 0.03) and role difficulties (*p* = 0.04) scores, was found to be statistically significant.

The increase in the scores of general health (*p* = 0.04), general vision (*p* = 0.0001), and near activities (*p* = 0.0001) as ETDRS visual acuity values increased was found to be statistically meaningful.

The ETDRS V.A. between ≤20 and 41–50 groups were found to be statistically significant in ocular pain (*p* = 0.01) and distance activities (*p* = 0.01) scores.

The general health (*p* = 0.04), general vision (*p* = 0.001), and near activities (*p* = 0.008) were found to be significantly high in the group without CSC, in comparison to the group with CSC.

The general health (*p* = 0.03), general vision (*p* = 0.0001), and near activities (*p* = 0.0001) scores were significantly high in the group without CSME in comparison to the group with CSME.

### 3.6. Conclusion

Stepwise linear regression analysis has been carried out in order to determine the factor or factors that have the highest effect on the questionnaire scores from all the factors that were found to be statistically significant during univariate analysis. The results of this analysis can be seen in Table 9.

The most effective factors were found to be* diabetics duration term* in the general health score (*p* = 0.029);* DRP stage* (*p* = 0.011) and* CSME presence* (*p* = 0.019) in general vision score;* near vision acuity* (*p* = 0.0001) and* DRP stage* (*p* = 0.020) in near activities score;* Snellen V.A.* (*p* = 0.014) in distance activities score;* ETDRS V.A.* in role difficulties (*p* = 0.034) and visual dependency (*p* = 0.011) scores; and Snellen V.A. (*p* = 0.026) in total scores.

## 4. Discussion

### 4.1. The General Health Scores

Klein et al. have examined the relationship between the NEI-VFQ-25 subscale and total scores in relation with vision acuity, DRP stage, and other demographic and clinical characteristics among patients with type 1 diabetes whose ages were averaged between 40.6 ± 10.7. The average general health scores of the patients in the study have been calculated as 60.5 ± 23.8 [15]. In our study the mean general health scores have been found to be worse than Klein et al. and Cusick et al.'s studies.

Cusick et al. have examined the relationship between the NEI-VFQ-25 near and far subscale scores with the central vision function (contrast sensitivity and visual acuity) in 170 patients with type 1 and type 2 diabetes patients [16]. The mean general health scores in this study have been given as 50.0 ± 2.0.

In our study the mean general health scores have been found to be 45.99 ± 16.46 in our study. Patients with any kind of chronic diseases that could affect their quality of living besides diabetes were not included in the study. However patients with complications due to diabetes and patients with hypertension were included within the scope of the study.

In our study it has been found that the general health scores was significantly lower in patients with hypertension in comparison with the patients without hypertension, and the factors related with the general health scores have also been examined being different to the other two studies (*p* = 0.039). Moreover it has been found out that the general health score decreased as the DRP stage increased (*p* = 0.04). It has also been found that the general health scores of the patients significantly decreased as ETDRS visual acuity values decreased (*p* = 0.04). It has been found that the general health score decreased again in the presence of CSC (*p* = 0.03) and CSME (*p* = 0.03). However it has been determined that the most effective factor in the general health score as a result of the multivariate analysis was the duration of the diabetes disease (*p* = 0.029). It is normal to find that the most important factor in general health score is the diabetes duration as we can imagine that as the duration of the disease increases the DRP stage and other systemic complications related with the disease also increase.

### 4.2. The General Vision Scores

The general vision scores of the patients included in the study of Klein et al. have been found to be 79.7 ± 16.1 [15]. In this study it has been found that the general vision score shows decrease in vision acuity due to increased age, diabetes duration, HbA1c levels, presence of hypertension, DRP stage, and decrease in the presence of macular edema and cataract presence. As a result of the multivariate analysis carried out in this study it has been found that the most important factor affecting the general vision was visual acuity.

In our study the general vision scores of the patients were found to be 53.96 ± 17.96. İt has been found that the general vision scores significantly decreased as the diabetes (*p* = 0.007), DRP stage (*p* = 0.0001), and HbA1c levels (*p* = 0.04) increased. We have also determined that the general vision scores significantly decreased again as the need to use insulin in order to control the blood sugar levels increased (*p* = 0.04).

It has been determined following the multivariate analysis that the most important factors on general vision scores were DRP stage (*p* = 0.011) and CSME (*p* = 0.019).

Similar to the other two studies, in our study we have also found that the general vision score was the lowest score following the general health score. As DRP patients have lower visual acuity or they are worried that they will have low visual acuity even though they do not have at the moment, it is normal for the general vision score to be low. The reason that the general vision score is lower than distance acuity and near vision scores related to vision could be that the difficulty degree that forms the general vision subscale score is not questioned but it is completely left to the comments of the patients; thereby said patients could be generally reflecting their worries regarding vision.

### 4.3. The Color Vision, Peripheral Vision, and Ocular Pain Scores

The color vision score of the patients according to the study of Klein et al. has been given as 94.8 ± 14.9, ocular pain score as 92.6 ± 13.0, and peripheral vision score as 89.3 ± 20.9 [15]. The ocular pain score of the patients in the study of Cusick et al. has been given as 93 ± 1.1, color vision score as 90.0 ± 1.6, and peripheral vision score as 82.0 ± 2.0 [15]. In both studies these three scores form the highest scores amongst all scores.

In our study, the color vision score (92.04 ± 17.71) has also been given as the highest score. The average ocular pain score (77.77 ± 20.54) and peripheral vision score (77.98 ± 25.44) have also been determined as the highest scores in our study. We believe that these scores have been found to be high because as long as glaucoma does not develop in patients with diabetes ocular pain is not frequently seen and color vision and peripheral vision defects are not frequently faced and because said symptoms are not given too much importance by the patients in comparison to loss of vision. Our pain scores are less than other studies, because our patients did not have glaucoma. Dry eye problems are also prevalent in this population. It could also be cultural differences.

### 4.4. The Near Vision Activities Scores

In our study, the mean near vision activities of the patients have been determined to be 75.71 ± 23.28. As the DRP stages increased a significant level of decrease has been noted in the near activities scores (*p* = 0.0001). Moreover it has been determined that the near activities score significantly decreased as the Snellen (*p* = 0.0001) V.A. and ETDRS V.A. (*p* = 0.0001) levels decreased. It has been found out that a significant decrease was obtained in the near activities scores within the presence of CSCat (*p* = 0.01) and CSME (*p* = 0.0001). It has been found out that the most effective factors that affected near activities scores as a result of the multivariate analysis were near vision acuity (*p* = 0.0001) and DRP stage (*p* = 0.020). The decrease in near activities (*p* = 0.0001) was statistically meaningful among the increased DRP stages in our study. However as a result of the multivariate analysis it has been decided that the near vision acuity value in near activities scores was more effective in comparison to the DRP stage.

### 4.5. The Distance Activities Scores

We attribute the changes of the distance activities scores between only these two age groups to the fact that the transition from 51–60 years to 61–70 years age group also symbolizes the transition from said patients from a more active social life to a less active period. Significant decrease has been determined in distance activities scores as DRP stages increased between stage 1 DRP group (*p* = 0.0001) where DRP was not found and stage 4 DRP group (*p* = 0.005) where DRP was not found. Moreover as Snellen V.A. values decrease it was found that distance activities score significantly decreased also (*p* = 0.04). A significant difference has been determined in distance activities scores between ETDRS V.A. values of ≤20 and 41–50 groups (*p* = 0.01). As a result of the multivariate analysis however we found out that the most effective factor in distance activities scores was the Snellen V.A. value (*p* = 0.014). In our study the significant decrease in Snellen (*p* = 0.0001) and ETDRS (*p* = 0.0001) V.A. was statistically meaningful among increasing DRP stages in our study. However it has been determined during the multivariate analysis that the Snellen V.A. value was more effective on the distance activities score in comparison to the DRP stage.

### 4.6. The Mental Health Scores

It has been determined in the study of Klein et al. that a significant level of decrease was seen on vision specific mental health when the diabetes duration term was high, the HbA1c level was high, hypertension was present, there was a decrease in visual acuity, DRP stages increased, and macular edema and cataract were present [15]. As a result of the multivariate analysis, it has been found however that the most effective factor in mental health was the visual acuity. In this study, the most effective factors on the social functioning, role difficulties, and visual dependency scores were not carried out.

In our study age has not been determined as an effective factor on vision specific social functioning score. However we determined that the vision specific mental health (*p* = 0.04), role difficulties (*p* = 0.04), and dependency (*p* = 0.009) scores among groups between the ages of 51–60 and 61–70 decreased significantly with increasing age. We believe that the patients passed onto an inactive period from a social and functional period during their passage from the 51–60 age group to the 61–70 age group and that the difficulty related to vision during this period caused these patients to be dependent on others and as a result we believe that the these patient's mental health was impaired.

We believe that the decrease in the visual acuities of patients must lead to difficulty in carrying out their daily activities and that this must lead to them being dependent on others. However we believe that the decrease in the visual acuities of patients does not affect their social relationships within a social community or their mental health. This could be arising from the beliefs, fatalism, and acceptance understanding of the people in our community.

### 4.7. Total Scores

The study of Klein et al. has determined a significant decrease in total scores (TS) with increased age, high HbA1c levels, the presence of hypertension, reduction in visual acuity, the increase in DRP stage, and the presence of macular edema and cataract [15]. A directly proportional decrease in the total questionnaire score has been found in this study with the decrease in visual acuity after characteristics such as age, retinopathy level, and other clinical features are checked.

In our study the decrease in total scores between the ages of 51–60 and 61–70 was significantly meaningful (*p* = 0.028). The decrease in TS was significant as the DRP stage increased between stage 1 and stage 0 DRP groups, (*p* = 0.0001), between stage 4 and stage 0 DRP groups (*p* = 0.001), and between stage 3 and stage 4 DRP groups (*p* = 0.001). It has been found out that the most effective factor in TS following multivariate analysis was the Snellen V.A. value (*p* = 0.026).

Broman et al. have used the NEI-VFQ-25 scale in the study they carried out on 4774 people aged over 40 living in Arizona, and they have examined the relationship between the decrease of visual acuity and various eye diseases on life quality dependent on vision [17].

In the study where Knudtson et al. examined the life qualities of 2670 patients, between the years of 1998–2000, who had eye diseases in relation to age such as senile macular degeneration, cataract, DRP, glaucoma, and macular edema, when other factors were also checked, it has been found out that the reduction in visual acuity led to the decrease of the quality of life [22].

In the study carried out on 5119 patients with cataract, not corrected refractive disorders, glaucoma, and senile macular degeneration, by Nirmalan et al., after demographic and clinical factors were checked, it has been observed that life quality and visual function scores were related with the visual acuity of the eye that sees better [23].

It has been stated that the slight decrease of 4.7 in the ETDRS value in the study of Cusick et al. had led to a 25-point decrease in near vision activities subscale scores (*p* ≤ 0.001) [16]. In the same study, it has been stated that the slight decrease in the ETDRS value has led to a 25-point decrease in the distance activities subscale scores (*p* ≤ 0.01).

In the study where Hariprasad et al. compared quality of vision and vision-specific quality of life in type 2 diabetes patients with macular oedema versus patients with type 1 diabetic retinopathy. They concluded that type 2 diabetes patients with macular oedema experience a decreased vision-specific quality of life compared with type 1 diabetic patients with diabetic retinopathy, glaucoma, or cataracts. However, vision-specific quality of life in type 2 diabetic patients with macular oedema was similar to those individuals with age related macular degeneration [24].

In the other study Gabrielian et al. compared vision related quality of life between patients with nonproliferative diabetic retinopathy (NPDR) and proliferative diabetic retinopathy (PDR). They found that VFQ is a superior measure of vision-specific quality of life for patients with diabetic retinopathy because it better captures mental and emotional aspects of the disease as well as visual function. Subjects with PDR versus NPDR suffer significant loss of vision-specific quality of life [25].

In our study, as a result of the multivariate analysis, it has been found out that the most effective factor in distance activities score (*p* = 0.014) and total score (*p* = 0.026) was* Snellen visual acuity*, that the most effective factor in near activities score was* near vision visual acuity* (*p* = 0.0001), and that the most effective factor in role difficulties (*p* = 0.034) and visual dependency (*p* = 0.011) was* ETDRS visual acuity*.

In conclusion, we can state that the vision related quality of life in type 2 diabetes patients at different DRP stages is related actually to visual acuity rather than stages and other demographic and clinical factors and that NEI-VFQ-25 is an efficient questionnaire that can evaluate the life quality of patients depending on vision. NEI-VFQ questionnaire can be used to measure the efficiency of interventions regarding treatment in the ophthalmology field, to the quality of life of the patients.

## Figures and Tables

**Table 1:** Age and gender distribution of patients according to stages.

	(*n* = 65)	(*n* = 34)	(*n* = 26)	(*n* = 28)	(*n* = 48)	
Gender						
Female	38	20	12	13	20	0.51^b^
Male	27	14	14	15	28	
Age (year)	61.09 ± 10.91	61.29 ± 10.61	59.80 ± 8.77	59.96 ± 7.35	58.22 ± 8.96	0.56

^a^One-way ANOVA, ^b^chi-square.

**Table 2:** Distribution of the hypertension presence of patients according to stages.

	Stage 0	Stage 1	Stage 2	Stage 3	Stage 4	*p* ^a^
	(*n* = 65)	(*n* = 34)	(*n* = 26)	(*n* = 28)	(*n* = 48)	
HTN (*n*)						
Absent	24	11	13	8	19	0.53
Present	41	23	13	20	29	

^a^Chi-square.

**Table 3:** Clinical characteristics of patients in relation to diabetes according to stages.

		Stage 0	Stage 1	Stage 2	Stage 3	Stage 4	*p* ^a^
		(*n* = 65)	(*n* = 34)	(*n* = 26)	(*n* = 28)	(*n* = 48)	
Diabetes duration (year)		8.65 ± 7.77	14.22 ± 7.90	13.96 ± 9.57	16.96 ± 7.25	16.77 ± 7.63	*0.0001*

Blood sugar control (*n*)	Diet	6	1	0	0	0	*0.002* ^b^
	OAD	33	8	8	9	14	
	Insulin	25	25	18	19	34	

PPG		136.60 ± 43.61	169.29 ± 67.90	142.19 ± 54.10	170.00 ± 58.20	144.32 ± 46	*0.01*

*HbA1c*		*6.44 ± 1.00*	*7.43 ± 1.50*	*8.32 ± 1.99*	*7.73 ± 1.33*	*7.11 ± 1.50*	*0.0001*

PPG: preprandial blood glucose, OAD: oral antidiabetic, ^a^one-way ANOVA, and ^b^ *t*-test.

**Table 4:** Ophthalmologic findings of patients according to stages.

		Stage 0	Stage 1	Stage 2	Stage 3	Stage 4	*p* ^a^
Snellen		0.95 ± 0.10	0.95 ± 0.08	0.80 ± 0.24	0.66 ± 0.28	0.59 ± 0.29	0.0001

ETDRS		55.75 ± 6.59	53.64 ± 6.39	47.84 ± 9.93	42.64 ± 12.81	37.70 ± 17.32	0.0001

Near vision		0.99 ± 0.02	1 ± 0	0.87 ± 0.25	0.86 ± 0.26	0.74 ± 0.32	0.0001

IOP		14.72 ± 2.33	14.50 ± 3.73	15.11 ± 2.08	14.21 ± 2.29	14.62 ± 2.23	0.77

Cataract	Operation (*n*)	4	6	4	2	12	

CSME	CSCat − (*n*)	56	22	16	22	24	0.01^b^
	CSCat + (*n*)	5	6	6	4	12	
	Present (*n*)	0	4	11	12	22	0.000^b^
	Absent (*n*)	65	30	15	16	26	

Near vision: near vision acuity; IOP: intraocular pressure; CSCat: clinically significant cataract (+: present −: not present).

CSME: clinically significant macular edema; ^a^one-way ANOVA; ^b^ *t*-test.

**Table 5:** NEI-VFQ-39 subscale and total scores of patients.

	NEI-VFQ-39 subscale and total scores		
	Minimum	Maximum	Average
General health	5.00	87.50	45.99 ± 16.46
General vision	10.00	100.00	53.96 ± 17.96
Ocular pain	12.50	100.00	77.77 ± 20.54
Near activities	4.16	100.00	75.71 ± 23.28
Distance activities	4.16	100.00	79.58 ± 21.46
Social functioning	12.50	100.00	88.64 ± 19.15
Mental health	0.00	100.00	70.47 ± 27.97
Role difficulties	0.00	100.00	69.72 ± 29.32
Visual dependency	0.00	100.00	84.47 ± 26.75
Driving	0.00	100.00	62.02 ± 31.14
Color vision	25.00	100.00	92.04 ± 17.71
Peripheral vision	25.00	100.00	77.98 ± 25.44
Total score	19.12	99	76.39 ± 18.63

**(a):** 

	*n*	General health	General vision	Ocular pain	Near activities	Distance activities	Social functioning	Mental health
Age								
≤50	32	50.85 ± 17.31	57.10 ± 20.88	73.28 ± 22.93	74.91 ± 28.90	77.07 ± 18.28	90.23 ± 16.28	65.62 ± 28.47
51–60	78	44.58 ± 16.76	52.08 ± 19.10	78.68 ± 19.85	74.00 ± 23.26	83.26 ± 17.40	92.14 ± 13.66	74.72 ± 25.43
61–70	60	47.70 ± 16.09	54.66 ± 14.89	78.95 ± 20.77	76.98 ± 22.73	74.02 ± 27.46	83.77 ± 25.83	63.75 ± 31.80
≥71	31	41.20 ± 14.41	54.11 ± 17.49	77.82 ± 19.55	78.38 ± 18.09	83.67 ± 18.54	87.63 ± 17.26	77.82 ± 22.60
*p* ^a^		0.08	0.59	0.60	0.79	0.04	0.07	0.03
Gender								
Female	103	43.98 ± 16.20	54.22 ± 17.77	73.30 ± 20.63	75.76 ± 23.30	81.49 ± 20.49	90.49 ± 16.54	72.04 ± 28.20
Male	98	48.11 ± 16.55	53.69 ± 18.24	82.47 ± 19.45	75.66 ± 23.38	77.57 ± 22.36	86.70 ± 21.47	68.83 ± 27.77
*p* ^b^		0.07	0.83	0.001	0.97	0.19	0.16	0.41
HTN								
Present	126	44.10 ± 16.11	52.93 ± 17.72	79.86 ± 20.24	77.71 ± 22.09	81.26 ± 19.62	90.18 ± 17.27	72.01 ± 26.83
Not present	75	49.16 ± 16.68	55.70 ± 18.34	74.26 ± 20.68	72.36 ± 24.95	76.77 ± 24.11	86.05 ± 21.84	67.89 ± 29.80
*p* ^b^		0.03	0.29	0.06	0.11	0.15	0.16	0.31

**(b):** 

	*n*	Role difficulties	Visual dependency	Driving	Color vision	Peripheral vision	Total score
Age							
≤50	32	62.10 ± 29.69	85.74 ± 23.07	41.66 ± 35.35	91.40 ± 18.63	78.12 ± 24.38	74.29 ± 17.41
51–60	78	76.12 ± 25.07	89.67 ± 22.81	69.26 ± 33.43	94.55 ± 15.93	81.08 ± 23.89	79.91 ± 14.84
61–70	60	63.22 ± 34.27	75.47 ± 32.96	54.16 ± 29.24	89.83 ± 19.76	71.66 ± 28.17	71.15 ± 22.96
≥71	31	74.03 ± 25.01	87.50 ± 22.47	76.18 ± 20.65	90.51 ± 16.92	82.25 ± 23.44	79.82 ± 17.16
*p* ^a^		0.02	0.01	0.16	0.43	0.12	0.02
Gender							
Female	103	72.33 ± 27.84	85.20 ± 27.70	64.26 ± 31.40	93.38 ± 16.47	78.64 ± 24.10	77.65 ± 18.03
Male	98	66.97 ± 30.70	83.71 ± 25.83	58.79 ± 31.37	90.62 ± 18.93	77.29 ± 26.88	75.06 ± 19.24
*p* ^b^		0.19	0.69	0.57	0.27	0.70	0.32
HTN							
Present	126	71.33 ± 27.76	85.45 ± 25.82	63.50 ± 30.89	92.33 ± 17.21	78.96 ± 24.98	77.33 ± 17.87
Not present	75	67.00 ± 31.77	82.82 ± 28.34	59.16 ± 32.51	91.55 ± 18.63	76.33 ± 26.28	74.79 ± 19.85
*p* ^b^		0.32	0.50	0.66	0.76	0.47	0.35

HTN: hypertension; ^a^one-way ANOVA; ^b^ *t*-test.

**(a):** 

	*n*	General health	General vision	Ocular pain	Near activities	Distance activities	Social functioning	Mental health
Time (year)								
≤10	90	48.19 ± 17.10	58.33 ± 17.61	78.13 ± 21.45	78.46 ± 22.21	79.34 ± 21.18	87.79 ± 20.36	70.23 ± 27.85
11–14	26	45.38 ± 14.10	53.46 ± 19.48	76.44 ± 20.41	78.18 ± 24.73	83.00 ± 21.45	93.74 ± 12.97	77.88 ± 21.12
15–19	30	44.58 ± 18.78	52.66 ± 20.72	76.16 ± 20.32	73.58 ± 25.03	77.94 ± 24.41	87.63 ± 21.42	61.16 ± 30.86
≥20	55	43.45 ± 14.99	47.77 ± 14.32	77.04 ± 19.65	71.21 ± 23.16	79.17 ± 20.61	88.18 ± 18.35	72.45 ± 28.68
*p* ^a^		0.36	0.007	0.95	0.27	0.81	0.54	0.14
Stage								
0	65	49.26 ± 16.83	62.88 ± 16.37	76.84 ± 21.39	86.01 ± 15.71	87.33 ± 14.98	93.77 ± 11.67	80.28 ± 19.11
1	34	48.89 ± 16.66	62.64 ± 13.14	77.20 ± 18.06	84.77 ± 13.65	65.64 ± 26.41	78.18 ± 25.76	53.27 ± 31.96
2	26	45.38 ± 13.97	49.61 ± 14.43	81.25 ± 19.76	6.31 ± 21.13	77.19 ± 21.23	87.88 ± 23.06	69.46 ± 30.96
3	28	44.73 ± 13.99	47.94 ± 15.36	80.80 ± 19.38	72.94 ± 23.82	89.68 ± 10.48	95.53 ± 08.01	88.03 ± 13.00
4	48	40.57 ± 17.45	41.61 ± 16.95	75.78 ± 22.39	56.64 ± 26.34	74.37 ± 23.37	85.50 ± 20.87	59.68 ± 29.28
*p* ^a^		0.04	0.0001	0.74	0.0001	0.0001	0.0001	0.0001
HbA1c								
>8	25	44.10 ± 16.87	51.20 ± 14.86	80.50 ± 19.12	81.36 ± 15.63	82.26 ± 21.47	91.99 ± 15.30	76.60 ± 22.76
≤8	75	48.00 ± 16.39	58.60 ± 16.45	79.26 ± 18.95	83.41 ± 18.05	80.47 ± 22.57	88.77 ± 19.36	69.81 ± 29.15
*P* ^b^		0.30	0.04	0.77	0.61	0.72	0.45	0.29
Treatment								
Diet	7	54.28 ± 09.54	63.57 ± 09.88	66.07 ± 21.30	80.46 ± 22.53	80.93 ± 12.92	90.46 ± 07.49	64.34 ± 23.43
OAD	72	48.57 ± 16.90	57.08 ± 19.96	78.57 ± 20.34	78.88 ± 22.69	79.85 ± 21.52	88.67 ± 19.72	73.69 ± 27.40
Insulin	121	43.73 ± 16.06	51.50 ± 16.70	72.78 ± 20.57	73.35 ± 23.57	79.32 ± 22.01	88.49 ± 19.45	68.88 ± 28.66
*p* ^a^		0.05	0.04	0.30	0.24	0.97	0.96	0.43

^a^one-way ANOVA and ^b^ *t*-test.

**(b):** 

	*n*	Role difficulties	Visual dependency	Driving	Color vision	Peripheral vision	Total score
Time (year)							
≤10	90	70.90 ± 28.77	84.93 ± 26.50	64.08 ± 26.75	91.94 ± 16.26	78.33 ± 26.01	76.22 ± 18.12
11–14	26	75.04 ± 29.00	89.34 ± 24.49	55.0 ± 51.23	95.83 ± 15.92	79.80 ± 28.30	79.85 ± 18.09
15–19	30	64.37 ± 31.10	81.45 ± 28.59	76.18 ± 21.74	91.66 ± 22.10	70.83 ± 26.32	74.14 ± 19.46
≥20	55	68.18 ± 29.61	83.06 ± 27.52	52.26 ± 33.55	90.74 ± 18.36	80.45 ± 22.40	76.24 ± 19.51
*p* ^a^		0.54	0.70	0.42	0.70	0.38	0.72
Stage							
DRP	65	82.50 ± 20.22	93.93 ± 16.00	76.91 ± 20.16	95.56 ± 12.41	85.00 ± 22.00	83.05 ± 13.57
1	34	52.24 ± 32.59	64.13 ± 36.64	62.96 ± 35.13	88.97 ± 19.64	61.76 ± 25.55	63.96 ± 22.08
2	26	71.15 ± 28.26	81.97 ± 23.34	37.49 ± 05.89	92.30 ± 18.39	77.88 ± 23.79	76.93 ± 18.80
3	28	80.58 ± 23.34	95.31 ± 12.45	77.77 ± 25.45	98.21 ± 06.55	90.17 ± 17.13	85.84 ± 09.14
4	48	57.68 ± 30.94	81.11 ± 29.44	50.24 ± 33.92	85.93 ± 23.58	72.91 ± 28.16	70.35 ± 19.59
*p* ^a^		0.0001	0.0001	0.10	0.012	0.0001	0.0001
HbA1c							
>8	25	75.25 ± 29.78	88.00 ± 23.31	51.66 ± 45.79	94.00 ± 18.08	78.00 ± 26.33	80.50 ± 17.38
≤8	75	70.76 ± 30.83	81.91 ± 28.68	69.60 ± 27.78	93.15 ± 15.17	76.66 ± 26.74	75.96 ± 19.27
*p* ^b^		0.52	0.33	0.44	0.81	0.82	0.29
Treatment							
Diet	7	70.53 ± 28.80	83.92 ± 22.78	72.22 ± 12.72	89.28 ± 13.36	85.71 ± 13.36	73.86 ± 11.85
OAD	72	71.09 ± 29.13	85.51 ± 26.43	62.72 ± 30.39	92.60 ± 18.12	80.20 ± 25.50	76.83 ± 19.03
Insulin	121	68.70 ± 29.74	83.76 ± 27.39	60.14 ± 33.89	91.80 ± 17.85	76.23 ± 25.99	76.21 ± 18.87
*p* ^b^		0.86	0.90	0.82	0.87	0.41	0.91

**(a):** 

	*n*	General health	General vision	Ocular pain	Near activities	Distance activities	Social functioning	Mental health
Snellen								
≤0.2	8	38.75 ± 14.94	35.93 ± 20.26	76.56 ± 20.52	46.56 ± 31.98	62.03 ± 23.87	79.15 ± 30.12	46.87 ± 31.27
0.3–0.5	32	41.64 ± 17.76	40.62 ± 15.74	72.65 ± 26.64	53.40 ± 25.51	75.09 ± 24.04	84.03 ± 23.15	64.21 ± 29.37
0.6–0.8	39	45.44 ± 11.75	46.73 ± 14.01	85.25 ± 14.01	73.05 ± 20.87	80.45 ± 17.73	89.84 ± 17.09	76.28 ± 25.04
>0.8	122	47.78 ± 17.30	60.96 ± 15.79	76.80 ± 20.08	84.33 ± 16.38	81.63 ± 21.24	90.09 ± 17.63	71.81 ± 27.55
*p* ^a^		0.15	0.0001	0.057	0.0001	0.04	0.19	0.02
ETDRS								
≤20	11	34.31 ± 14.49	31.13 ± 17.65	64.77 ± 28.94	39.54 ± 32.69	64.24 ± 25.74	82.94 ± 25.16	49.54 ± 29.53
21–40	29	42.75 ± 17.83	42.75 ± 16.34	77.15 ± 23.40	60.57 ± 24.87	72.94 ± 22.18	84.68 ± 23.15	68.10 ± 25.96
41–50	49	46.88 ± 14.43	49.59 ± 15.95	84.43 ± 16.04	73.39 ± 21.17	83.05 ± 19.35	90.81 ± 19.39	72.63 ± 28.81
>50	112	47.58 ± 16.73	61.02 ± 15.24	76.29 ± 19.91	84.20 ± 16.30	81.29 ± 21.00	89.28 ± 17.21	72.20 ± 27.43
*p* ^a^		0.04	0.0001	0.01	0.0001	0.01	0.39	0.06
Cataract								
CSCat −	140	47.85 ± 16.77	56.89 ± 17.74	77.64 ± 20.35	78.67 ± 22.48	80.26 ± 20.45	89.93 ± 17.36	71.57 ± 25.86
CSCat +	33	40.22 ± 14.96	44.54 ± 17.52	76.51 ± 21.59	65.50 ± 22.23	77.91 ± 23.71	85.02 ± 21.98	67.12 ± 33.28
Operated	28	43.48 ± 15.14	50.44 ± 15.44	79.91 ± 20.78	72.96 ± 25.41	78.15 ± 24.19	86.45 ± 23.75	68.92 ± 31.92
*p* ^a^		0.03	0.001	0.80	0.01	0.79	0.33	0.68
CSME								
Not present	152	47.40 ± 16.70	57.82 ± 17.06	78.09 ± 20.38	79.76 ± 20.52	80.37 ± 21.09	88.92 ± 18.39	70.34 ± 28.36
Present	49	41.63 ± 15.03	41.98 ± 15.31	76.78 ± 21.19	63.14 ± 26.83	77.15 ± 22.62	87.78 ± 21.52	70.89 ± 27.01
*p* ^b^		0.03	0.0001	0.70	0.0001	0.36	0.71	0.90

*p*: one-way ANOVA; ^b^ *t*-test CSCat: clinically significant cataract.

**(b):** 

	*n*	Role difficulties	Visual dependency	Driving	Color vision	Peripheral vision	Total score
Snellen							
≤0.2	8	46.87 ± 38.95	72.65 ± 43.42	75.00 ± 35.35	87.50 ± 26.72	65.62 ± 35.19	64.14 ± 24.73
0.3–0.5	32	61.71 ± 27.98	79.88 ± 25.13	48.43 ± 37.12	88.28 ± 20.06	77.34 ± 25.68	72.45 ± 19.30
0.6–0.8	39	74.03 ± 25.91	90.01 ± 18.63	49.35 ± 30.13	90.13 ± 19.74	80.76 ± 23.27	77.91 ± 15.91
>0.8	122	71.93 ± 29.32	84.68 ± 27.89	73.80 ± 25.58	93.95 ± 15.54	78.07 ± 25.42	77.73 ± 18.62
*p* ^a^		0.03	0.24	0.06	0.28	0.50	0.12
ETDRS							
≤20	11	49.43 ± 34.39	73.29 ± 39.83	50.00 ± 70.71	79.54 ± 26.96	68.18 ± 35.51	64.47 ± 24.35
21–40	29	63.57 ± 27.70	82.47 ± 23.09	53.4 ± 29.74	90.51 ± 20.50	75.86 ± 25.42	72.67 ± 17.57
41–50	49	71.68 ± 28.55	87.48 ± 25.32	53.70 ± 34.38	93.75 ± 15.89	85.20 ± 20.99	79.20 ± 18.27
>50	112	72.44 ± 28.92	84.77 ± 26.78	70.83 ± 26.69	92.95 ± 16.28	76.33 ± 25.74	77.29 ± 18.10
*p* ^a^		0.05	0.43	0.32	0.09	0.09	0.07
Cataract							
CSCat −	140	71.79 ± 27.87	86.21 ± 25.33	29.77 ± 5.62	93.29 ± 16.69	79.10 ± 24.47	77.28 ± 17.41
CSCat +	33	66.66 ± 31.11	80.05 ± 29.02	29.12 ± 10.29	90.62 ± 18.78	75.75 ± 26.13	74.83 ± 19.79
Operated	28	62.94 ± 33.76	81.02 ± 30.78	40.82 ± 14.43	87.50 ± 20.97	75.00 ± 29.65	73.74 ± 23.05
*p* ^a^		0.28	0.37	0.92	0.25	0.63	0.57
CSME							
Not Present	152	70.19 ± 29.18	84.48 ± 26.03	61.10 ± 31.15	91.94 ± 17.74	77.79 ± 25.33	76.49 ± 18.39
Present	49	68.23 ± 29.99	84.43 ± 29.15	64.77 ± 32.45	92.34 ± 17.82	78.57 ± 26.02	76.07 ± 19.53
*p* ^b^		0.68	0.99	0.74	0.89	0.85	0.89

**Table 9:** Stepwise linear regression analysis of the questionnaire scores.

Score	*R*	*R* ^2^	*p*	Related factor
General health	0.219	0.048	0.029	Duration

General vision	0.447	0.200	0.019	Duration *p* = 0.011 CSME *p* = 0.019

Near activities	0.536	0.287	0.038	Near V.A. *p* = 0.0001 Stage *p* = 0.020

Distance activities	0.244	0.060	0.014	Snellen V.A.

Role difficulties	0.212	0.045	0.034	ETDRS V.A.

Visual dependency	0.253	0.064	0.011	ETDRS V.A.

Total score	*0.223*	*0.05*	*0.026*	Snellen V.A.
